# Effects of lifestyle changes and high-dose β-blocker therapy on exercise capacity in children, adolescents, and young adults with hypertrophic cardiomyopathy

**DOI:** 10.1017/S1047951114000237

**Published:** 2014-03-10

**Authors:** Ewa-Lena Bratt, Ingegerd Östman-Smith

**Affiliations:** 1 Department of Paediatrics, Institute of Clinical Sciences, The Sahlgrenska Academy, University of Gothenburg, Gothenburg, Sweden; 2 Department of Paediatric Cardiology, The Queen Silvia Children’s Hospital, Gothenburg, Sweden

**Keywords:** Exercise capacity, hypertrophic cardiomyopathy, β-receptor antagonist, propranolol, metoprolol

## Abstract

*Aim:* The use of β-blocker therapy in asymptomatic patients with hypertrophic cardiomyopathy is controversial. This study evaluates the effect of lifestyle changes and high-dose β-blocker therapy on their exercise capacity. *Methods and results:* A total of 29 consecutive newly diagnosed asymptomatic patients with familial hypertrophic cardiomyopathy, median age 15 years (range 7–25), were recruited. In all, 16 patients with risk factors for sudden death were treated with propranolol if no contraindications, or equivalent doses of metoprolol; 13 with no risk factors were randomised to metoprolol or no active treatment. Thus, there were three treatment groups, non-selective β-blockade (n=10, propranolol 4.0–11.6 mg/kg/day), selective β-blockade (n=9, metoprolol 2.7–5.9 mg/kg/day), and randomised controls (n=10). All were given recommendations for lifestyle modifications, and reduced energetic exercise significantly (p=0.002). Before study entry, and after 1 year, all underwent bicycle exercise tests with a ramp protocol. There were no differences in exercise capacity between the groups at entry, or follow-up, when median exercise capacity in the groups were virtually identical (2.4, 2.3, and 2.3 watt/kg and 55, 55, and 55 watt/(height in metre)^2^ in control, selective, and non-selective groups, respectively. Maximum heart rate decreased in the selective (−29%, p=0.04) and non-selective (−24%, p=0.002) groups. No patient developed a pathological blood-pressure response to exercise because of β-blocker therapy. Boys were more frequently risk-factor positive than girls (75% versus 33%, p=0.048) and had higher physical activity scores than girls at study-entry (p=0.011). *Conclusions:* Neither selective nor non-selective β-blockade causes significant reductions in exercise capacity in patients with hypertrophic cardiomyopathy above that induced by lifestyle changes.

Hypertrophic cardiomyopathy is an inherited cardiac disease with an estimated prevalence in the adult population of 1:500.^1^ ^,^ ^2^ It is characterised by left ventricular hypertrophy and is the most common cause of sudden cardiac death during exercise in childhood and adolescence.^3^ ^,^ ^4^ The highest risk of sudden cardiac death caused by hypertrophic cardiomyopathy is in the 8–16-year age range.^5^ Clinical evaluation at diagnosis should include family history, echocardiography, electrocardiogram, ambulatory electrocardiogram Holter monitoring, and exercise stress testing in order to assess risk for sudden death. Risk stratification based on these findings plays an important role in directing treatment.^6^ ^–^ ^8^ An important part of risk stratification is to assess the physiologic response to exercise in order to evaluate blood pressure response and arrhythmias during exercise. Hypotensive blood pressure response during exercise is associated with adverse long-term prognosis.^9^ ^,^ ^10^ A failure of systolic blood pressure to increase by at least 20 mmHg from rest to peak exercise or a progressive decrease in blood pressure during exercise is regarded as an abnormal response and a risk factor for sudden cardiac death in adults,^6^ ^,^ ^10^ and failure-related death in children.^11^

Previous studies suggest that restriction from competitive sports results in lower mortality rates,^12^ as does high-dose β-blocker treatment in a dose-dependent manner, at least in childhood hypertrophic cardiomyopathy.^13^ ^–^ ^15^ It is important that patients with hypertrophic cardiomyopathy receive information regarding restriction of such physical activity that is thought to increase the risk of sudden death.^9^ ^,^ ^16^ ^,^ ^17^ Therapy with β-blockers continues to be at the frontline of medical therapies for children and adults with hypertrophic cardiomyopathy.^8^ ^,^ ^11^ ^,^ ^15^ ^,^ ^18^ ^,^ ^19^ However, the use of high doses of β-blockers is controversial, particularly in asymptomatic patients, because of the concerns about the presumed side effects such as impairment of exercise tolerance.

To the best of our knowledge, there are no published studies that have assessed the effect of lifestyle changes and high-dose β-blockers on exercise capacity in patients with hypertrophic cardiomyopathy. Neither are there any long-term studies, and long-term therapy may confer more benefit on diastolic function in hypertrophic cardiomyopathy patients than the acute adreno-receptor blocking effect.^20^ The purpose of this study was to evaluate exercise capacity in patients with hypertrophic cardiomyopathy before, and 1 year after, start of high-dose β-blocker therapy and advice on lifestyle changes, and to compare them with contemporary controls with mild hypertrophic cardiomyopathy without β-blocker treatment, but who are recommended the same lifestyle changes.

## Materials and methods

### Study groups

Asymptomatic patients with hypertrophic cardiomyopathy were consecutively recruited from January, 2005 to December, 2010 as part of a prospective family screening study of familial hypertrophic cardiomyopathy at the Queen Silvia Children’s Hospital, Gothenburg, Sweden. Patients had to be at least 125 cm tall to be able to use the ergometer bicycle. Patients up to 25 years of age were included in the study. A total of 30 patients fulfilled these inclusion criteria and all agreed to participate in the study. However, one patient was excluded because of newly diagnosed hypothyroidism associated with marked obesity – body mass index of 37.

**Figure 1:**
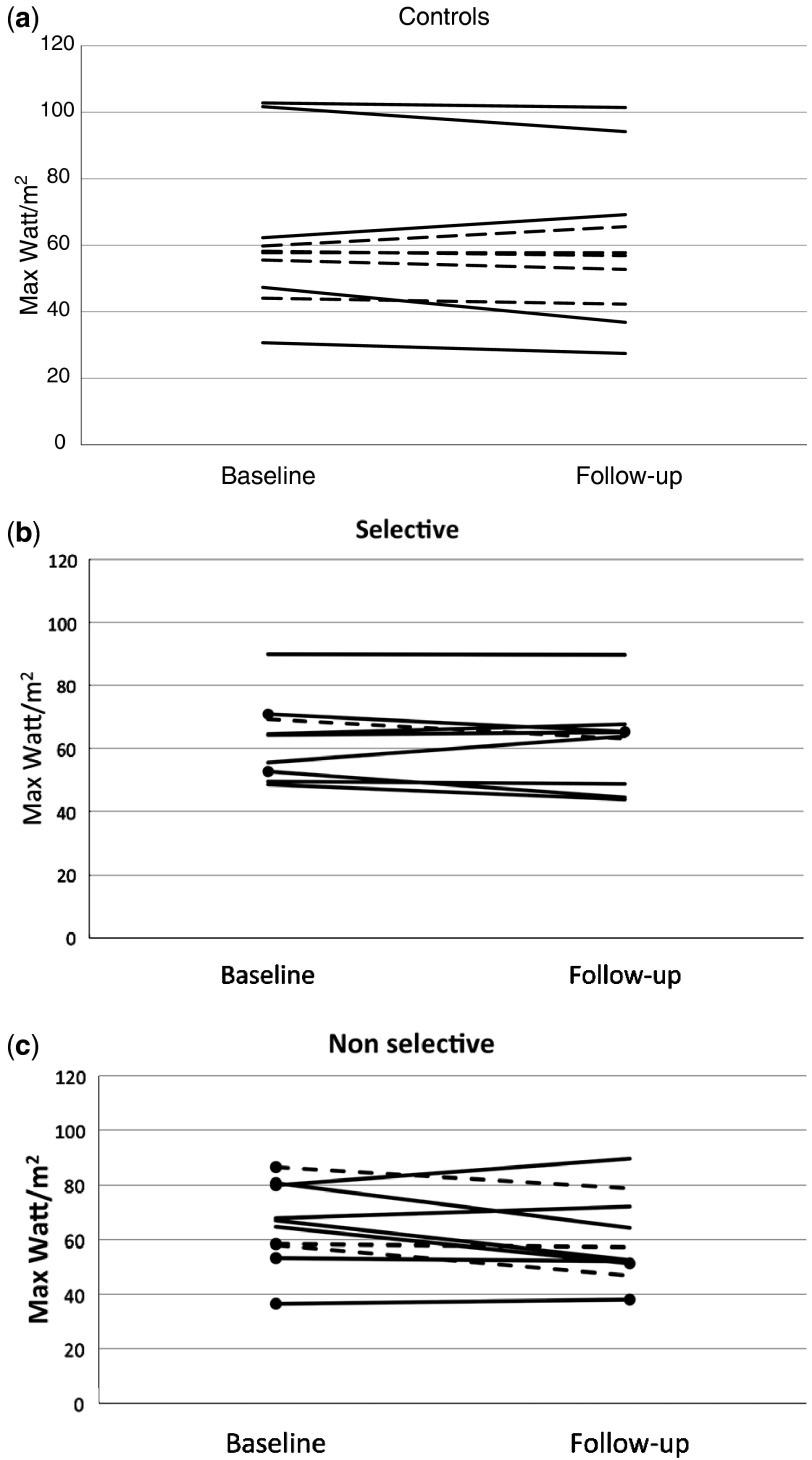
(***a***) Exercise capacities in watt/(height in metre)^2^ at baseline and at follow-up in the control group (hypertrophic cardiomyopathy patients treated with lifestyle modifications only), girls are indicated by a dotted line, boys by a solid line. (***b***) Exercise capacities in watt/(height in metre)^2^ at baseline and at follow-up in the hypertrophic cardiomyopathy group treated with selective β-blocker therapy. Round filled dots at the end of the lines indicate those patients who had a pathological blood pressure response during or after exercise test at baseline, or at follow up. (***c***) Exercise capacities in watt/(height in metre)^2^ at baseline and at follow-up in the hypertrophic cardiomyopathy patients treated with non-selective β-blocker therapy. Round filled dots at the end of the lines indicate those patients who had a pathological blood pressure response during or after exercise test at baseline, or at follow up. In all, four out of six of patients with initially pathological response had a normalised blood pressure response on therapy.

All 29 individuals had a complete risk assessment. The highest risk for sudden death has been associated with the presence of any of the following: previous cardiac arrest, non-sustained ventricular tachycardia noted on Holter electrocardiogram, pathological blood pressure response to exercise, particularly severe cardiac hypertrophy, family history of sudden cardiac death related to hypertrophic cardiomyopathy, and malignant electrocardiogram pattern.^7^ ^,^ ^10^ ^,^ ^14^ ^,^ ^21^ Thus, positive risk factor screening was defined as the presence of at least one of the risk factors listed in Table 1.^7^ ^,^ ^14^ ^,^ ^21^ Patients who were risk factor positive were recommended treatment with propranolol, a non-selective β-blocker (n=10, “non-selective” group), or if there were contraindications to this, such as bronchial asthma, a selective β-blocker (metoprolol, n=6). Only 1 out of 29 patients had left ventricular outflow obstruction, which was very mild at rest (outflow velocity of 2 m/second), but increased to a Doppler-predicted gradient of 64 mmHg after Valsalva. This, however, did not interfere with exercise capacity, as this patient had among the highest exercise capacity of all patients before treatment was commenced. He was treated with propranolol because of a close family history of sudden death (see Fig 1c). There was one high-risk patient – who had additional psychosocial stress – who developed central side effects on propanolol, which persisted on metoprolol and was therefore converted to atenolol (3.7mg/kg/day) plus disopyramide. This patient is not analysed in the non-selective group – on intention to treat criteria – but as he had received a selective β-blocker for the last 9 months before the follow-up study he is analysed together with the metoprolol-treated patients (n=6). Patients with no risk factors (n=13) were randomised either to follow-up, without pharmacological treatment (n=10, control group), or to selective β-blocker therapy with metoprolol (n=3). The two subgroups receiving metoprolol – or atenolol – were added together to form the “selective” group. High-dose propranolol therapy in children has been defined as a propranolol dose ⩾5 mg/kg,^14^ or equivalent doses of other β-blockers; however, there are not only substantial inter-individual variation in the rates of hepatic metabolism of β-blockers, there are great age-related variations in the dose required to give the same plasma levels, with young children demanding substantially higher dose in mg/kg than teenagers, and teenagers needing higher dose than adults for the same plasma levels.^15^ ^,^ ^22^ It is therefore scientifically more correct to standardise the dose on the degree of physiological β-blockade than on dose in mg/kg. Thus, although the target dose of β-blocker was 6 mg/kg/day for propanolol, or a minimum of 4.0 mg/kg/day for metoprolol, with sufficient β-blockade on 24-hour Holter monitoring – for Holter criteria of adequate β-blockade see references^13^ ^,^ ^15^, a few of the young adults needed lower doses for a profound β-receptor blockade to be evident on Holter and exercise testing. The randomised patients were seen 3-monthly during the first year of therapy, with a standardised rate of drug therapy increase, with assessment of patient compliance to both exercise restrictions and drug therapy; 24-hour Holter recordings were always carried out after 9 months therapy on approximately – as dictated by tablet/capsule size – 4.5 mg/kg propranolol equivalent dose in order to assess whether β-blockade was sufficient, or a last increase to around 6 mg/kg was necessary. High-risk patients had additional earlier Holter recordings to assess β-blockade and absence of arrhythmias, and often required higher doses to achieve a good heart rate control according to criteria.^13^ ^,^ ^15^ The evidence that this approach was successful is in the similar degree of reduction in exercise heart rate response seen in all patients on β-blockers.

**Table 1:** Risk factors for sudden death used as criteria for group selection.

• Family history of sudden death in a close relative younger than 30 years with hypertrophic cardiomyopathy.
• Syncope related to exertion.
• Pathological blood pressure response during or after exercise test defined as failure of systolic blood pressure to rise by at least 25 mmHg from baseline values, or as a fall in systolic blood pressure.
• Non-sustained or sustained ventricular tachycardia noted on Holter registration.
• Maximal wall thickness >190% of the 95th centile prediction limits for age.
• Electrocardiogram amplitude QRS-sum in limb leads more than 12 mV or electrocardiogram risk score 6 or above.^14^ ^,^ ^21^

All patients received the same recommendations regarding participation in competitive and recreational sports activities following the guidelines published by the American Heart Association and the European Society of Cardiology.^16^ ^,^ ^17^

### Exercise protocol

Before study entry, and after 1 year, all patients underwent incremental bicycle (Monark ergomedic 839E) exercise tests with a ramp protocol starting at 1 watt/kg body weight, with 10 watts increments each minute. The exercise test was performed with the patient in a sitting position. We monitored 12-lead electrocardiogram (Welch Allyn Cardio Perfect 1.5.0.434), systolic blood pressure – manual blood pressure measurements – and respiratory rate every minute before, during and a minimum of 15 minutes after exercise. This software also converts recorded exercise load into assessed Metabolic Equivalents of Task.

### Resting electrocardiogram

QRS-amplitude voltage sums were added in all six limb-leads as described previously,^13^ ^,^ ^23^ and electrocardiogram-risk score was calculated according the criteria published earlier.^21^

### Echocardiography

Echocardiographic measurements of left ventricular wall thickness was carried out by long-axis M-mode echocardiography, and related to 95th centile prediction limits as described previously.^13^ A maximal wall thickness exceeding 190% of the 95th centile prediction limit was considered a risk factor (corresponds to Z-score >3.72).^14^

### Questionnaire regarding compliance to lifestyle recommendations

Of the 29 patients, 23 also completed and returned a questionnaire regarding the frequency of strenuous physical activities – defined as becoming exhausted – sports participation, and leisure time activities, such as spending time with friends, using a computer, watching TV, visiting the cinema, theatre, or playing or listening to music, before diagnosis and after 1 year. The questions taken, and scoring used, were from a validated Quality of Life questionnaire.^24^ The physical activity grade was scored in one of five levels. The individuals returning the questionnaires were evenly distributed between the groups – 8, 7, and 8 responders in the respective groups. The questionnaire was filled out as a complete self-report or together with the parents depending on age. To those patients who did not respond after the first request, we sent a reminder letter after 4 weeks.

### Statistical analysis

Statistical analysis was carried out using commercial software (PASW statistics 18.0). The non-parametric Kruskal–Wallis test was used for intergroup comparisons and the Wilcoxon signed test was used for paired comparisons within groups, with each patient serving as his own control. Fisher’s two-tailed exact test was used to compare proportions. Spearman’s rank correlation was used to analyse correlations between the dose of β-blocker and change in exercise capacity from baseline to follow-up. The scores from the questionnaire were analysed using the Wilcoxon signed-rank test, and irregular distributions compared using the Kolmogorov–Smirnov test (Statgraphics Plus v.5.2).

## Results

### Characteristics of the patients

There were no significant differences in age, baseline electrocardiogram, or echocardiographic variables between the groups (Table 2). There was a male preponderance with a male to female ratio of 20:9, as is common in this age-range of hypertrophic cardiomyopathy patients. An uneven gender distribution between the groups was noted (controls 5/10), selective group 8/9 and non-selective group 7/10 males, but this did not reach statistical significance. This was partly caused by the fact that boys were risk-factor positive in a significantly higher proportion than girls, 75% versus 33% (p=0.048), and thus were more likely to end up with active treatment.

**Table 2:** Clinical characteristics of patient groups.

	Control group (n=10)	Selective group (n=9)	Non-selective group (n=10)	p-value (Kruskal–Wallis)
Age	14.5 (11.0–18.0)	13.0 (11.5–15.3)	13.0 (11.5–15.3)	0.667
QRS-LL sum	6.13 (5.7–8.3)	5.95 (4.8–8.7)	7.13 (5.1–9.5)	0.788
SEPPER	118 (112–130)	123 (113–167)	143 (119–184)	0.269
LVPER	92.5 (81–109)	111 (103–120)	106 (96–116)	0.116

Age=age in years; LVPER=posteriour left ventricular wall thickness expressed in per cent of the 95th centile prediction limit for posterior left ventricle wall; QRS LL sum=QRS-limb lead voltage amplitude sum; SEPPER=septal thickness expressed in per cent of the 95th centile prediction limit for septal thickness; Values given as median (interquartile range)

All 29 patients completed the exercise test at baseline and at follow-up after a median of 12 months’ continuous therapy/exercise restriction (range 11–13). The 10 patients included in the non-selective group were all treated with propranolol. The median β-blocker dose at follow-up was 5.8 mg/kg/day (range 4–11.6) in the non-selective β-blocker group and 5.3 mg/kg/day (range 2.7–6.91) in the selective β-blocker group – atenolol dose converted to metoprolol equivalents.

### Exercise capacity

As the study groups included growing children and adolescents, the exercise capacity was related both to body weight and body height. In agreement with Döbeln and Eriksson,^25^ we found a closer correlation between work capacity and height squared (correlation coefficient r=0.935) than for work capacity versus body weight (correlation coefficient r=0.872) or for work capacity versus height (correlation coefficient r=0.929), even though the difference between watt/metre height and watt/metre^2^ was small. In the Tables, we have accordingly given the data both as the conventional watt/kg body weight and in watt/metre.^2^ For ease of comparison with treadmill studies, we have also given the data in Metabolic Equivalent of Task in Table 4. Body mass index did not differ between groups, and did not change significantly from baseline to follow-up (Table 3). Most of the patients were still growing, and thus both height and weight tended to increase over time (Table 3).

**Table 3:** Changes in BMI, height and weight.

	Control group	Selective group	Non-selective group	p-value (baseline versus follow-up)		
	(n=10)	(n=9)	(n=10)	Control	Selective	Non- selective
BMI baseline	21 (19–22)	22 (18–26)	22 (18–26)	0.754	0.109	0.109
BMI follow-up	21 (20–24)	24 (20–28)	24 (20–28)			
Height baseline	162 (147–170)	163 (147–176)	163 (147–176)	0.008	0.039	0.039
Height follow-up	169 (154–173)	167 (154–180)	167 (154–180)			
Weight baseline	54 (43–63)	70 (42–75)	70 (42–75)	0.021	0.002	0.002
Weight follow-up	58 (49–67)	75 (48–80)	75 (48–80)			

BMI=body mass index; Height=height in centimetres; Weight=weight in kilogram Values given as median (interquartile range).

There were no significant differences in exercise capacity either in watt/kg (p=0.7), in watt/metre^2^ (p=0.2), or in Metabolic Equivalents (p=0.6) between treatment groups at entry into the study. Boys had a higher work capacity than girls when expressed as watt/kg, median 2.68 (range 2.1–3.8) versus 2.36 (2.1–2.7; p=0.007) and in Metabolic Equivalents, median 10.9 (range 10.0–12.2) versus 10.2 (range 9.3–11.4; p=0.01) but the difference was not significant when expressed as watt/metre^2^, boys median 63.3 (30.7–80.6) versus 57.9 (44.1–59.8; p=0.320).

Neither were there any significant differences between treatment groups in exercise capacity at follow-up (Table 4). The only patient who had a small resting outflow-gradient before treatment had his gradient disappear with β-blocker therapy. The work capacity of each patient, at diagnosis, and after 1 year of treatment, is illustrated in Fig 1a–c. The change from baseline to follow-up was not significant within any of the groups, see Table 4, and quite small. Indeed, 2 out of 10 of the controls, 3 out of 9 in the selective group, and 3 out of 10 in the non-selective group actually improved their performance at follow-up. Median work capacity was virtually identical at follow-up in all three groups, both related to weight and to body height (Table 4). In boys, the median change in watt/metre^2^ was −5.1%, and the two individuals with the greatest percentage fall in exercise capacity were both in the control group (see Fig 1a). In girls, the median change in exercise capacity was −2.4%, and changes at follow-up were very small for most (Fig 1a–c).

**Table 4:** Exercise capacity.

	Control group	Selective group	Non-selective group	p-value (baseline versus follow-up)		
	(n=10)	(n=9)	(n=10)	Control	Selective	Non- selective
Watts/m^2^ baseline	58 (47–72)	66 (57–80)	66 (57–81)	0.344	0.344	0.344
Watts/m^2^ follow-up	55 (38–64)	55 (50–74)	55 (50–74)			
Watts/kg baseline	2.7 (2.5–3.0)	2.6 (2.1–3.1)	2.6 (2.1–3.2)	0.289	0.109	0.109
Watts/kg follow-up	2.4 (1.9–2.8)	2.3 (2.2–2.6)	2.3 (2.2–2.6)			
METs baseline	10.3 (9.7–12.0)	10.5 (9.7–11.4)	10.1 (8.4–11.7)	0.070	0.109	0.109
METs follow-up	10.3 (8.8–11.0)	9.8 (8.9–9.8)	9.2 (8.6–10.1)			
HR max baseline	184 (168–199)	184 (163–194)	182 (176–187)	1	0.039	0.002
HR max follow-up	184 (177–188)	129 (123–156)	138 (119–144)			
SBP max baseline	154 (129–166)	152 (127–173)	152 (127–172)	0.508	0.754	0.754
SBP max follow-up	161 (138–185)	139 (126–177)	139 (126–177)			
SBP increase baseline	43 (35–56)	38 (22–63)	38 (22–63)	0.021	1.000	1.000
SBP increase follow-up	52 (36–67)	36 (13–64)	36 (13–64)			

HR=heart rate; kg=kilogram body weight; m^2^=height in metre^2^; METs=metabolic equivalent of task; SBP=systolic blood pressure Results given as median (interquartile range)

There was a complete lack of significant correlation between the dose of β-blocker and the per cent change of exercise capacity (watt/metre^2^) from baseline to follow-up in both the selective (correlation coefficient −0.04, p=0.93) and in the non-selective (correlation coefficient −0.29; p=0.49) treatment groups.

The lifestyle changes with exercise restrictions were a factor common to all groups, and thus we also analysed all three groups combined. There was no significant change in body mass index (p=0.15) or in exercise capacity expressed as watt/metre^2^ (p=0.38), but there was a small but significant reduction in exercise capacity expressed as watt/kg (p=0.004). This might be influenced by the fact that the weight increase between baseline and follow-up tended to be between 2.2% and 4.6% greater than the height increase in all three groups (see Table 3).

### Heart rate and systolic blood pressure

There was no difference between maximal heart rate or maximal systolic blood pressure on exercise at baseline between the controls and the two treatment groups (Table 4). In the control group, there was no significant change in maximum heart rate response to exercise between baseline and follow-up (Table 4). However, in the groups treated with selective and non-selective β-blockers, there were statistically significant decreases of maximum heart rate, −29% in the selective group and −24% in the non-selective group (Table 4), between baseline and follow-up. Systolic blood pressure rise on exercise was not significantly different at follow-up compared with baseline in any group (Table 4) and no individual developed a pathological blood pressure response as a result of β-blocker therapy (Fig 1b–c). On the contrary, out of six patients with abnormal blood pressure response at diagnosis, four normalised their blood pressure response to exercise on propranolol therapy (Fig 1c).

### Maximum respiratory rate

There were no significant differences between the groups in maximum respiratory rate, reflecting the exercise effort, at baseline or at follow-up.

### Compliance to lifestyle recommendations

**Figure 2:**
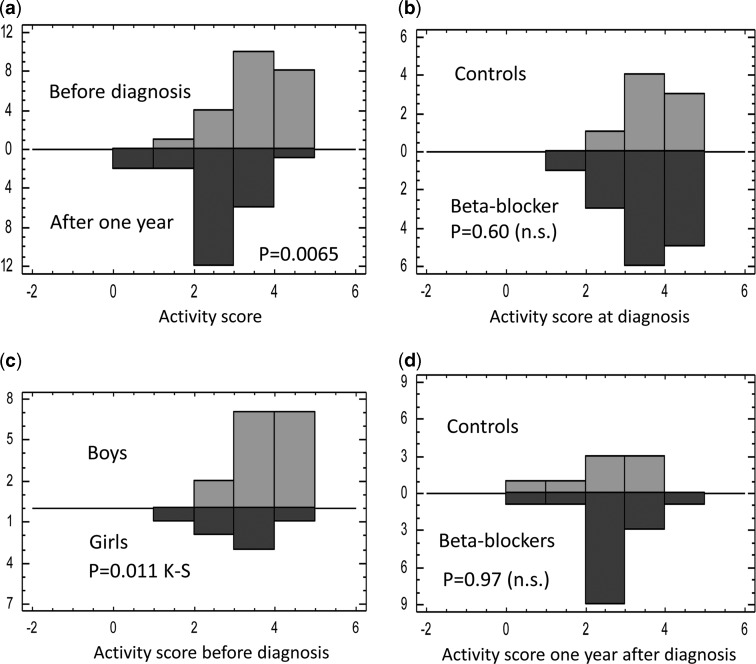
(***a***) Scores of time spent in intensive physical activity each week from patients in the study, before diagnosis (above line) as compared with after 1 year of follow-up (below line). Score range possible 0–5, the decrease within patients is significant (p=0.002). The distribution of scores is also different on the Kolmogorov–Smirnov test (p=0.0065). (***b***) Comparing activity score of intensive exercise activity each week before the diagnosis between controls (above line) and patients treated with β-blockers (below line). There is no difference in either distribution pattern or numerical scores. (***c***) Comparing activity score of intensive exercise activity each week before the diagnosis in boys (above the line) versus girls (below line); distribution is different on the Kolmogorov–Smirnov test (p=0.011). (***d***) Illustrates that the pattern of activity at follow-up is the same in the controls (above the line) as in the patients treated with β-blockers (below the line), p=0.97.

There was a significant decrease in the numerical scores of strenuous physical exercise from before diagnosis compared with 1 year later (p=0.002), see Fig 2a. Before diagnosis, 35% performed strenuous exercise >7 hours/week compared to 4% 1 year later (p=0.011). The proportion of patients who never participated in sports activities increased from 9% to 22%, and thus the distribution of activity patterns was also significantly different at follow-up (p=0.0065, Fig 2a). There was no difference in activity pattern at diagnosis between the control group and the β-blocker-treated groups at the start of the study (Fig 2b). However, there was a different distribution of activity pattern between boys and girls, with the majority of boys taking part in intensive exercise several times per week, leading to a significantly different activity pattern in boys compared with girls (p=0.011, Fig 2c). At follow-up, the activity pattern had altered to the same degree in controls and β-blocker-treated groups (p=0.97, Fig 2d).

No change was detected regarding leisure time activities. Time spent watching TV and using a computer did not increase significantly.

## Discussion

The aim of the present study was to evaluate the effect of high-dose selective or non-selective β-blocker therapy and lifestyle changes on exercise capacity in patients with hypertrophic cardiomyopathy. High-dose β-blocker therapy in childhood patients with hypertrophic cardiomyopathy decreases mortality.^13^ ^,^ ^14^ Its use in low-risk patients is more controversial, however, because of perceived effects of high-dose β-blocker therapy on the quality of life and exercise tolerance.

### General effects of β-blocker therapy

Some reviews speculate that β-blocker therapy may affect growth in young children but without substantiating such claims.^7^ ^,^ ^26^ There was no impairment of growth among our treated patients, and the incidence of side effects was low, and could be satisfactorily solved by change of β-blocker.

### Effect of β-blocker therapy on exercise capacity

Studies on exercise performance of healthy individuals after β-blocker therapy have shown a discrepancy between selective and non-selective therapy. Non-selective β-blocker therapy has been reported to result in a decrease of maximum physical exercise performance, whereas selective β-blocker therapy did not impair the physical exercise performance significantly as compared with placebo.^27^ ^–^ ^29^ However, non-selective β-blockers are better able to treat a pathological blood pressure response to exercise in patients with hypertrophic cardiomyopathy than selective β-blockers as seen in our patients also.^15^ ^,^ ^30^

In the present study, comparable reductions in maximal exercise heart rate occurred with both selective and non-selective β-blocker therapy indicating equivalent β-blockade. Nevertheless, there was no significant impairment of exercise performance in any of the groups with selective or non-selective β-blocker therapy compared with patients treated with lifestyle changes only. In the control group treated with lifestyle changes only, 8 out of 10 patients had a lower maximal exercise capacity at follow-up, exactly the same proportion as in the selective and non-selective groups (see Fig 1a–c). Thus, there was a significantly lower exercise capacity at follow-up only when all groups were combined. Changes in girls were very small (−2.4% median change), and the largest drops were seen in athletically active boys with high fitness levels before imposition of exercise restrictions. Even if it is not possible in this rather small study to reliably separate the effects on exercise capacity of lifestyle modifications from the effects of β-blocker treatment, the differential effect depending on gender suggests that lifestyle modifications, consisting of restricting energetic exercise such as soccer and ice hockey, by themselves inevitably have some effect on aerobic capacity and therefore exercise capacity, and are the main cause of the changes observed. That they did significantly influence the amount of energetic exercise was proven by our questionnaire.

### Possible mechanisms for maintaining cardiac output in spite of β-blockade in patients with hypertrophic cardiomyopathy

There are a few small studies on the effect of short-term β-blocker therapy on exercise performance in patients with mostly symptomatic hypertrophic cardiomyopathy with conflicting results. A reduced exercise capacity (but with less symptoms) for nadolol,^31^ or unchanged or even improved exercise tolerance with propranolol have been reported.^32^ ^,^ ^33^

The results of this study indicate that high-dose β-blocker therapy by itself does not impair physical exercise capacity to any significant degree in this specific group of patients, in spite of the fact that the maximum heart rates were significantly decreased by 27–29%. This finding is clearly not due to β-blocker therapy reducing outflow obstruction, as only one patient had dynamic outflow obstruction before therapy, and he actually had a high exercise capacity with the outflow obstruction untreated. One possible mechanism to explain the maintained exercise capacity could be an improvement in diastolic function leading to an increased stroke volume to compensate for the lower heart rate.

The majority of adult patients with hypertrophic cardiomyopathy have an exercise capacity that is lower than predicted.^34^ ^,^ ^35^ They are unable to increase their stroke volume during exercise, and stroke volume might even fall secondary to a decrease of atrial contribution to preload.^36^ ^–^ ^39^ Patients with abnormal blood pressure response to exercise have particularly marked reduction in stroke volume during exercise.^36^ ^,^ ^37^ ^,^ ^39^ Exercise aggravates the relative disproportion between duration of systole versus duration of diastole with disproportionate shortening of diastolic filling time.^40^ ^,^ ^41^ However, as therapy with propranolol has been reported to improve diastolic dysfunction in adult patients with hypertrophic cardiomyopathy,^41^ ^,^ ^42^ and will allow longer diastolic filling time, there are potential mechanisms for β-blocker therapy to improve stroke volume on exercise in hypertrophic cardiomyopathy. Indeed, the patients in this study were part of a larger study also, including patients too small to perform an exercise test, which showed significant improvement by β-blocker therapy in five measures of diastolic function.^20^ In patients with hypertrophic cardiomyopathy, the stroke volume is the major determinant of peak exercise capacity and is determined by left ventricular diastolic fillings characteristics.^43^ It has been suggested that the depressed left ventricular relaxation during exercise in hypertrophic cardiomyopathy patients results from adrenergic stimulation.^44^ Thus, it is not surprising that β-blocker therapy may have beneficial effects on stroke volume during exercise.

### β-Blocker therapy and blood pressure response to exercise

As an important practical observation, this study indicates that β-blocker therapy does not reduce systolic blood pressure response to exercise sufficiently to give a false positive result during risk stratification.

### Limitations of the study

A limiting factor in this study is the modest number of patients included and the non-significantly skewed gender distribution with more girls in the control group. Therefore, our statistical comparisons have focused on within-patient changes. It cannot be excluded that β-blocker treatment in high doses had a slight negative effect on exercise capacity that was too small to be detected in a study of our size. Owing to the fact that it would be unethical to withhold internationally accepted advice on lifestyle changes to some patients, it was not possible to absolutely separate the effects on exercise capacity of lifestyle modifications from the effects of β-blocker treatment. However, although the patient numbers are limited, it is a uniquely homogeneous patient group, all newly diagnosed, and therefore not previously treated either pharmacologically or with lifestyle modifications. This has given us the opportunity to also assess the effect of training restriction on the exercise capacity of the control patients, and the effect of these restrictions on their activity pattern. Thus, we think this study gives guidance as to the very small size of effects on exercise capacity, if any, to be expected in young patients with hypertrophic cardiomyopathy treated with β-blockers in a high dose.

## Conclusion

Neither selective nor non-selective β-blockade by itself caused a significant reduction in exercise capacity in patients with hypertrophic cardiomyopathy, above that induced by lifestyle changes, in spite of a significant reduction of maximum heart rate response. One likely mechanism behind this finding is an improvement in diastolic filling resulting in an increase of previously compromised stroke volume during exercise.
